# Association of triglyceride-glucose index and its combination with obesity indicators in predicting the risk of aortic aneurysm and dissection

**DOI:** 10.3389/fnut.2024.1454880

**Published:** 2024-10-23

**Authors:** Wangqin Yu, Xiaoling Wang, Zhongyan Du, Wenke Cheng

**Affiliations:** ^1^School of Basic Medical Sciences, Zhejiang Chinese Medical University, Hangzhou, China; ^2^Department of Pharmacy, Lintong Rehabilitation and Recuperation Centre, Xi'an, China; ^3^Zhejiang Key Laboratory of Blood-Stasis-Toxin Syndrome, Zhejiang Engineering Research Center for "Preventive Treatment" Smart Health of Traditional Chinese Medicine, School of Basic Medical Sciences, Zhejiang Chinese Medical University, Hangzhou, China; ^4^Zhejiang Key Laboratory of Blood-Stasis-Toxin Syndrome, Zhejiang Chinese Medical University, Hangzhou, China; ^5^Medical Faculty, University of Leipzig, Leipzig, Germany

**Keywords:** TyG, TyG-BMI, TyG-WC, TyG-WHtR, aortic aneurysm and dissection, UK biobank

## Abstract

**Background:**

The association between the triglyceride-glucose (TyG) index and its combination with obesity indictors in aortic aneurysm and dissection (AAD) remains unclear. We aimed to investigate the association between TyG and TyG-body mass index (TyG-BMI), TyG-waist circumference (TyG-WC), TyG-waist height ratio (TyG-WHtR) and AAD risk.

**Methods:**

This study included 387,483 baseline participants from the UK Biobank with complete data on TyG, TyG-BMI, TyG-WC and TyG-WHtR. Cox proportional hazard models evaluated the relationship between these four indicators and the risk of AAD occurrence. Restricted cubic spline (RCS) examined the non-linear relationship between these indicators and AAD risk, while receiver operating characteristic (ROC) curves assessed the predictive value of these four indicators for AAD risk.

**Results:**

Over a median follow-up of 13.7 years, 3,041 AAD events were recorded. Multivariate Cox regression analysis indicated that for each standard deviation increase, the risk of AAD occurrence increased by 33% (HR: 1.33, 95%CI: 1.29–1.38), 25% (HR: 1.25, 95%CI: 1.21–1.29), 61% (HR: 1.61, 95%CI: 1.56–1.66) and 44% (HR: 1.44, 95%CI: 1.39–1.49) for TyG, TyG-BMI, TyG-WC and TyG-WHtR, respectively. RCS demonstrated a linear relationship between these indicators and AAD risk, with TyG-WC demonstrating the best performance in predicting AAD occurrence based on ROC curves.

**Conclusion:**

The present study, based on a large prospective cohort design, showed that higher TyG index and its combination with obesity indices were significantly associated with the risk of AAD. Moreover, AFT models further showed that elevation of these indicators significantly advanced the onset of AAD. In addition, RCS analyses demonstrated a linear association between these indicators and the risk of AAD, and the TyG-WC showed higher predictive ability for AAD. These findings emphasize the potential application of the TyG index and its combination with obesity indicators in the early identification of AAD.

## Introduction

Aortic aneurysm and dissection (AAD) poses a significant risk to cardiovascular health, with an extremely high mortality rate (1). Epidemiological data indicates an annual incidence of AAD ranging from 2 to 16 cases per 100,000 individuals, with a significant male predominance (2, 3). Statistics reveal that AAD claims over 150,000 lives annually (4). Currently, surgical intervention stands as the primary treatment, yet despite advancements, postoperative mortality rates persist above 10% (5). While medications like *β*-adrenergic receptor antagonists and angiotensin II receptor antagonists offer some control over aneurysm progression, the lack of precise biomarkers and effective therapeutic targets hampers prevention and treatment efforts (6, 7).

Previous research identifies smoking, hypertension, age and atherosclerosis as key AAD risk factors (8). Smoking is a well-established contributor, as it leads to chronic inflammation and weakening of the aortic wall, significantly increasing the risk of aneurysm formation (9). Hypertension, or high blood pressure, places additional stress on the aortic wall, which not only promotes the growth of aneurysms but also increases the risk of aortic dissection, where a tear in the inner layer of the aorta can occur (10). Age is another crucial factor, with the risk of both conditions increasing as the aorta becomes more fragile over time (11). Atherosclerosis, characterized by the accumulation of plaque in the arteries, can cause the stiffening and narrowing of the aorta, which further elevates the risk of aneurysm rupture and dissection by weakening the structural integrity of the arterial wall (12). Additionally, increasing evidence implicates diabetes and obesity in AAD development (13, 14). Insulin resistance (IR), a hallmark of diabetes and obesity, emerges as a pivotal contributor to various cardiovascular diseases (CVDs) (15). Furthermore, studies link higher IR markers with larger aneurysm diameters (16). IR disrupts metabolic processes and fuels inflammation, underscoring its potential significance in AAD onset and progression (17).

While the hyperinsulinemic-euglycemic clamp serves as the gold standard for IR measurement, its complexity including the need for specialized equipment, prolonged testing time, and skilled personnel limits its feasibility for routine clinical application (18). The triglyceride-glucose (TyG) emerges as a simpler, efficient alternative for early IR identification (19). Ahn et al. showed the potential efficacy of TyG in discerning prediabetes from diabetes in the general population (20). Moreover, it serves as a reliable indicator for various metabolic diseases, including stroke, CVD and metabolic syndrome (21–23). Furthermore, combining the TyG index with obesity indicators enhances diagnostic accuracy compared to TyG alone (24, 25).

To date, no study has explored the relationship between the TyG index, its combination with obesity indicators, and the risk of AAD. In summary, IR has been shown to increase the risk of AAD, and both the TyG index and obesity-related parameters hold promise as potential surrogates for IR. Therefore, we hypothesize that higher levels of the TyG index and its combinations with obesity parameters (TyG-BMI, TyG-WC, TyG-WHtR) are associated with an increased risk of AAD occurrence. In the present study, our aim was to investigate the associations between the TyG index, its combinations with obesity metrics, and the risk of AAD, as well as to compare the ability of these IR surrogates in predicting AAD occurrence.

## Methods

The UKB constitutes a large-scale, prospective, community-based cohort study aimed at advancing biomedical research and informing public health policies. Between 2006 and 2010, the project recruited over 500,000 participants from 22 centers across the United Kingdom. All participants were registered with the National Health Service (NHS), the publicly funded healthcare system in the United Kingdom, ensuring they had comprehensive health records available for long-term follow-up and research purposes. At baseline, participants completed detailed touchscreen questionnaires covering demographics, health and lifestyle factors, alongside undergoing physical examinations, functional assessments and providing blood, urine and saliva samples. Comprehensive study protocols and descriptions have been previously reported (26), and all data collection and research in the UKB adhere to strict ethical and privacy standards, with participants providing written informed consent before enrolment. The study received approval from the North West Multi-Center Research Ethics Committee and aligns with the Declaration of Helsinki principles.

### Ascertainment of exposures

Upon enrolment in the UKB, blood samples were randomly collected from participants with fasting times recorded prior to blood sampling. Given the logistic challenges of collecting fasting blood samples from a large, geographically dispersed population (27), biochemical measurements were conducted within 24 h on non-fasting serum samples, including triglycerides (TG), glucose, total cholesterol (TC), high (HDL)- and low-density lipoprotein cholesterol (LDL). The coefficients of variation for TG and glucose concentrations were both less than 3%. Physical measurements such as height, weight and WC were also ontained during baseline examinations. WHtR was computed as the ratio of WC to height (28). Four indices were calculated using the following formulas: TyG = ln [triglycerides (mg/dL) × glucose (mg/dL) / 2]; TyG-BMI = TyG * BMI; TyG-WC = TyG * WC; TyG-WHtR = TyG * WHtR (29, 30).

### Assessment of outcome

The diagnosis of AAD relied on the International Classification of Diseases, Tenth Revision (ICD-10) codes I71.0-I71.9, with data sourced from hospital admissions and death registries within the UKB. Participants were followed from the date of their recruitment into the study until the first of the following events occurred: a confirmed AAD event, death, or the study cutoff date of 26 October 2022. The follow-up period ended at whichever of these events happened first for each individual.

### Assessment of covariates

Baseline sociodemographic data encompassed sex, age, ethnicity, physical activity, smoking and drinking status, dietary habits, Townsend Deprivation Index (TDI), family history of CVD, medication usage and baseline history of chronic diseases, primarily cancer, heart diseases, hypertension and diabetes. Physical activity levels were quantified using weekly metabolic equivalent (MET) minutes (31). TDI reflected participants’ socioeconomic status at baseline (32). Dietary scores were derived from participants’ reports of consumption of nine food items, with detailed scoring information reported elsewhere (32, 33). Family history of CVD herein referred to parental heart disease history collected through self-reports at baseline. Medication history primarily encompassed antihypertensive drugs, lipid-lowering drugs and insulin.

### Selection criteria

**Figure 1:**
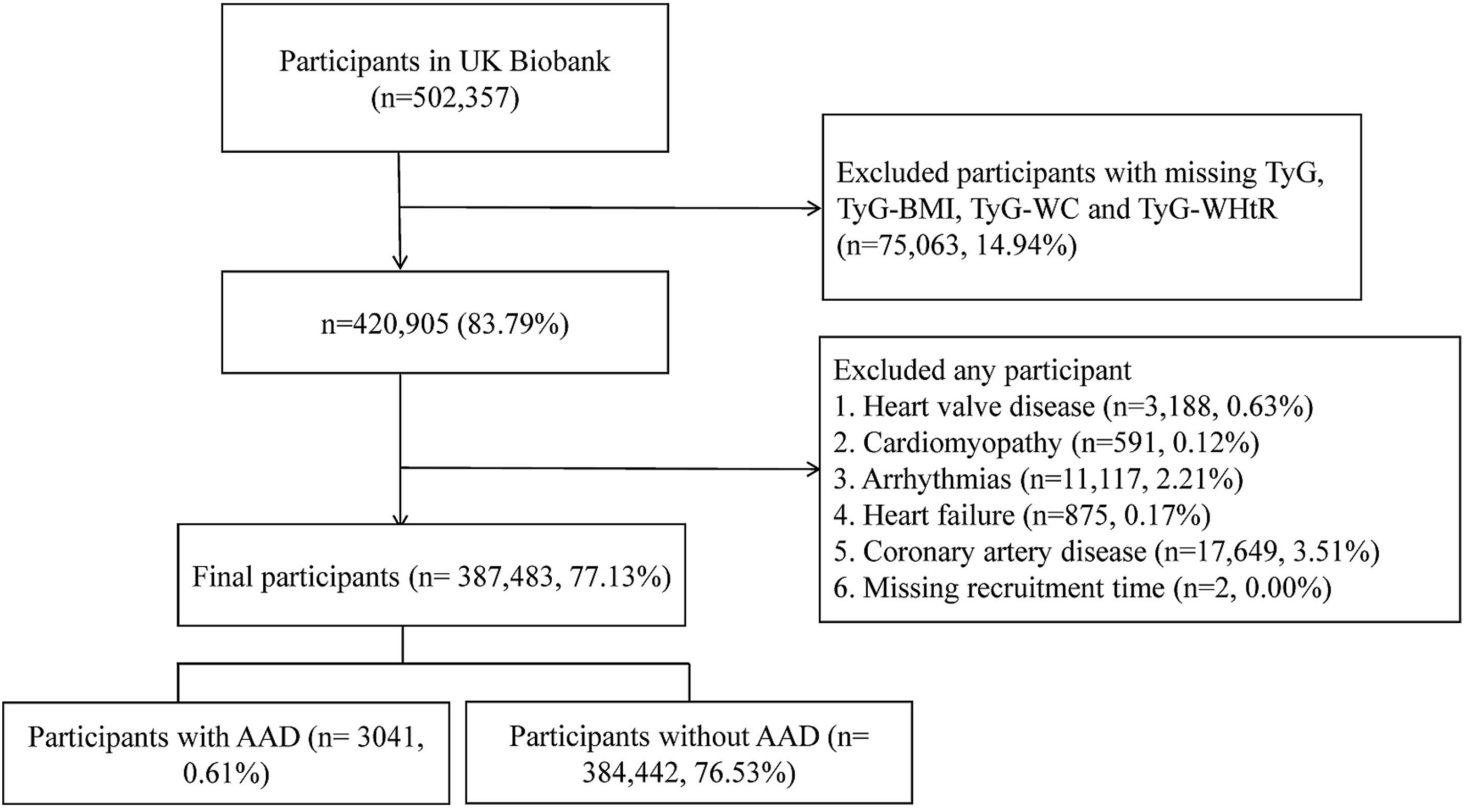
Flowchart of participant selection.

We initially included 502,357 participants from UK Biobank. We then excluded the participants with any missing TyG, TyG-BMI, TyG-WC and TyG-WHtR data (*n* = 75,063). Subsequently, we excluded two participants with missing recruitment time records. Additionally, to mitigate confounding effects, participants with baseline CVDs including heart valve disease, cardiomyopathy, arrhythmias, heart failure or coronary artery disease (*n* = 33,420) were further excluded (Supplementary Table S1). Consequently, a total of 387,483 participants were retained for subsequent analysis (Figure 1).

### Statistical analysis

Missing covariate values were imputed using multiple imputations via random forests, with one set of imputed data selected for analysis. Kolmogorov–Smirnov test was used to assess the distribution type of continuous variables. All continuous variables exhibited a skewed distribution. Baseline characteristics were stratified according to AAD and non-AAD groups, with continuous variables expressed as medians (interquartile ranges) and categorical variables presented as numbers and proportions (N, %). Comparisons between the two groups for continuous and categorical variables were conducted using the Mann–Whitney U test and Chi-square test, respectively Incidence of AAD across quartiles of TyG, TyG-BMI, TyG-WC and TyG-WHtR during the follow-up period was assessed using Kaplan–Meier (KM) curves, with significance evaluated by log-rank test. Cox proportional hazard models were employed to assess the association between TyG and its combination with obesity metrics and AAD risk across three Cox multivariable regression models. Model 1 lacked adjustments for any variables, while Model 2 adjusted for age, sex and race. Model 3 further adjusted for physical activity, smoking and drinking habits, diet score, TDI, fasting time, family history of CVD, usage of antihypertensive drugs, lipid-lowering drugs or insulin and baseline chronic diseases including cancer, hypertension and diabetes. The selection of confounders was determined using a directed acyclic graph (DAG)^1^ (34), with the results depicted in Supplementary Figure S1. Each model categorized TyG, TyG-BMI, TyG-WC and TyG-WHtR into quartiles, with the first quartile (Q1) serving as a reference to evaluate AAD risk trends and calculate *p*-values. Subsequently, these four metrics were standardized using Z-scores to assess AAD risk changes per one standard deviation (SD) increase.

Restricted cubic spline (RCS) analysis with three knots (10, 50, 90th) were employed to evaluate the non-linear association between these four indicators and AAD risk, adjusting for Model 3 covariates, with nonlinearity assessed using the log-likelihood ratio test. Subsequently, the accelerated failure time (AFT) model investigated the impact of TyG, TyG-BMI, TyG-WC and TyG-WHtR levels on AAD event timings (35). Using the lowest quartile group (Q1) as the reference, we assessed the effect of increases in these indices on the timing of AAD onset. Receiver operating characteristic (ROC) curves and area under the curve (AUC) analyses evaluated the diagnostic and predictive capabilities of the four indicators in predicting the risk of AAD. To assess the stability of the model, we randomly split the dataset into 70% training and 30% testing sets. We then plotted ROC curves for both sets to evaluate the model’s generalization ability on unseen data.

Additionally, subgroup analyses were conducted based on sex, age, BMI, smoking and alcohol consumption, fasting time, diet score, diabetes, hypertension, cancer, medication use and family history of CVD, with *p*-values for between-group interactions calculated via likelihood ratio tests. Finally, several sensitivity analyses were conducted to assess the robustness of our findings. (1) Participants who developed AAD within 2 years of the follow-up period were excluded to mitigate potential reverse causal effects. (2) Participants with any missing covariate values at baseline were excluded, and the main analysis was repeated. (3) To address the significant differences in sample sizes between the AAD and non-AAD groups, as well as differences in baseline characteristics, and avoid potential selection bias, propensity score matching (PSM) was employed in a 1:1 manner based on all Model 3 covariates. After calculating propensity scores, matching was performed using the nearest-neighbor matching algorithm with a caliper of 0.2 pooled SD (36). All analyses were performed using R (version 4.2.1), with statistical significance set at a two-sided *p*-value less than 0.05.

## Results

### Basic characteristics of participants

A total of 387,483 AAD-free participants, with a median age of 57 years and 44.6% males, were included. Table 1 illustrates baseline characteristics, categorized by AAD status. Compared to the non-AAD group, the AAD group exhibited higher age, BMI, WC, weight and height, alongside a higher proportion of male and white participants, and a higher prevalence of chronic diseases (all *p* < 0.001). Additionally, levels of TyG, TyG-BMI, TyG-WC and TyG-WHtR were significantly elevated in the AAD group compared to the non-AAD group (all *p* < 0.001).

**Table 1:** Baseline demographic and clinical characteristics.

Characteristic	Total	Non-AAD	AAD	*p*-value
	(*n* = 387,483)	(*n* = 384,442)	(n = 3,041)	
Age, years	57.0 (50.0–63.0)	57.0 (50.0–63.0)	63.0 (58.0–66.0)	<0.001
Male	172,711 (44.6%)	170,383 (44.3%)	2,328 (76.6%)	<0.001
White	366,889 (94.7%)	363,922 (94.7%)	2,967 (97.6%)	<0.001
Weight	76.0 (66.2–87.1)	76.0 (66.1–87.0)	83.0 (73.4–93.6)	<0.001
Height	168.0 (161.5–175.0)	168.0 (161.0–175.0)	173.0 (167.0–179.0)	<0.001
BMI	26.6 (24.1–29.7)	26.6 (24.1–29.7)	27.6 (25.1–30.6)	<0.001
WC	89.0 (80.0–98.0)	89.0 (80.0–98.0)	96.0 (88.5–104.0)	<0.001
MET	1800.0 (825.0–3573.0)	1800.0 (825.0–3572.0)	1815.0 (742.5–3767.5)	0.905
TDI	−2.2 (−3.7–0.4)	−2.2 (−3.7–0.4)	−2.1 (−3.6–0.6)	0.122
Fasting time	3.0 (2.0–4.0)	3.0 (2.0–4.0)	3.0 (3.0–4.0)	<0.001
Diet score	5.0 (4.0–6.0)	5.0 (4.0–6.0)	5.0 (4.0–6.0)	<0.001
DM	16,718 (4.3%)	16,547 (4.3%)	171 (5.6%)	<0.001
Hypertension	97,138 (25.1%)	95,802 (24.9%)	1,336 (43.9%)	<0.001
Cancer	34,108 (8.8%)	33,794 (8.8%)	314 (10.3%)	0.003
History of heart diseases family	149,648 (38.6%)	148,470 (38.6%)	1,178 (38.7%)	0.894
Lipid-lowering drugs	51,879 (13.4%)	51,056 (13.3%)	823 (27.1%)	<0.001
Antihypertensives	68,231 (17.6%)	67,187 (17.5%)	1,044 (34.3%)	<0.001
Insulin	3,363 (0.9%)	3,342 (0.9%)	21 (0.7%)	<0.001
Drinking status				<0.001
Never	16,552 (4.3%)	16,471 (4.3%)	81 (2.7%)	
Previous	12,995 (3.4%)	12,854 (3.3%)	141 (4.6%)	
Current	357,936 (92.4%)	355,117 (92.4%)	2,819 (92.7%)	
Smoking status				<0.001
Never	158,096 (40.8%)	157,362 (40.9%)	734 (24.1%)	
Previous	188,904 (48.8%)	187,306 (48.7%)	1,598 (52.5%)	
Current	40,483 (10.4%)	39,774 (10.3%)	709 (23.3%)	
TyG	8.7 (8.3–9.1)	8.7 (8.3–9.1)	8.8 (8.5–9.2)	<0.001
TyG-BMI	232.1 (203.7–265.7)	232.0 (203.6–265.6)	245.5 (217.3–277.8)	<0.001
TyG-WC	776.2 (676.1–878.6)	775.5 (675.5–877.9)	855.0 (763.0–948.5)	<0.001
TyG-WHtR	4.6 (4.1–5.2)	4.6 (4.1–5.2)	4.9 (4.4–5.5)	<0.001

AAD, aortic aneurysm and dissection; BMI, body mass index; WC, waist circumference; MET, metabolic equivalent task; TDI, Townsend deprivation index; DM, diabetes mellitus.

### The associations between TyG, TyG-BMI, TyG-WC, TyG-WHtR and the risk of AAD

**Figure 2:**
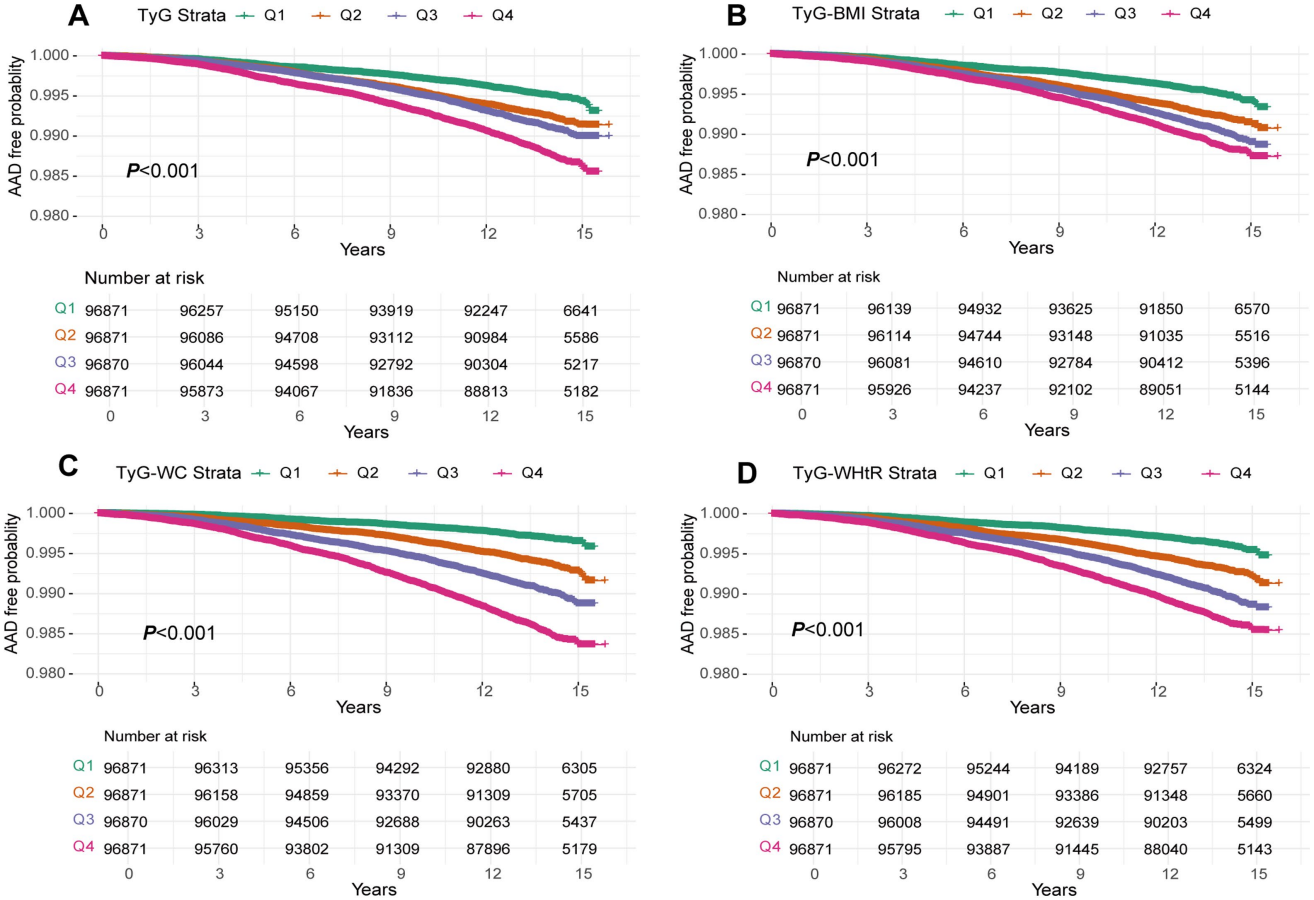
A Kaplan–Meier curves for AAD events in the TyG index **(A)**, TyG-BMI **(B)**, TyG-WC **(C)** and TyG-WHtR **(D)** quintile group. TyG index, triglyceride glucose index; TyG-BMI, triglyceride glucose index–body mass index; TyG-WC, triglyceride glucose index-waist circumference; TyG-WHtR, triglyceride glucose index-waist height ratio.

During a median follow-up of 13.7 years, 3,041 AAD cases were identified. KM curves illustrated an escalating risk of AAD with increasing quartiles of TyG, TyG-BMI, TyG-WC and TyG-WHtR (all *p*-values <0.001; Figure 2). Consistent findings emerged from Cox models. In Model 1, lacking adjustments, there was an upward trend in the relative risk of AAD occurrence with increasing quartiles of TyG, TyG-BMI, TyG-WC and TyG-WHtR (all P for trend <0.001; Table 2). For each SD increase, the risk of AAD occurrence increased by 33% (HR: 1.33, 95%CI: 1.29–1.38), 25% (HR: 1.25, 95%CI: 1.21–1.29), 61% (HR: 1.61, 95%CI: 1.56–1.66) and 44% (HR: 1.44, 95%CI: 1.39–1.49) for TyG, TyG-BMI, TyG-WC and TyG-WHtR, respectively. These associations persisted in Model 2 after adjusting for age, sex and race. Furthermore, in Model 3, adjusting for additional covariates, the increased quartiles of these four indicators enhanced the risk of AAD occurrence compared to the Q1 group, especially in the Q4 group. Meanwhile, each SD increase in these four indicators increased the risk of AAD occurrence by 10% (HR: 1.10, 95%CI: 1.05–1.14), 13% (HR: 1.13, 95%CI: 1.09–1.18), 21% (HR: 1.21, 95%CI: 1.16–1.26) and 15% (HR: 1.15, 95%CI: 1.10–1.19), respectively (Table 2).

**Table 2:** The association between TyG, TyG-BMI, TyG-WC, TyG-WHtR and the risk of AAD.

Type	Model 1		Model 2		Model 3	
	HR (95%CI)	*p*	HR (95%CI)	*p*	HR (95%CI)	*p*
TyG						
Q1	Reference		Reference		Reference	
Q2	1.51 (1.34–1.7)	< 0.001	1.14 (1.01–1.28)	0.033	1.1 (0.98–1.24)	0.111
Q3	1.77 (1.57–1.98)	< 0.001	1.15 (1.03–1.29)	0.017	1.08 (0.96–1.21)	0.198
Q4	2.39 (2.14–2.67)	< 0.001	1.43 (1.28–1.6)	< 0.001	1.29 (1.15–1.44)	< 0.001
*P* for trend	< 0.001		< 0.001		< 0.001	
Per SD increase	1.33 (1.29–1.38)	< 0.001	1.14 (1.1–1.18)	< 0.001	1.1 (1.05–1.14)	< 0.001
TyG-BMI						
Q1	Reference		Reference		Reference	
Q2	1.56 (1.39–1.76)	< 0.001	1.08 (0.96–1.22)	0.183	1.07 (0.95–1.2)	0.291
Q3	1.93 (1.72–2.16)	< 0.001	1.19 (1.06–1.33)	0.003	1.11 (0.99–1.25)	0.073
Q4	2.28 (2.04–2.54)	< 0.001	1.54 (1.37–1.72)	< 0.001	1.36 (1.21–1.53)	< 0.001
*P* for trend	< 0.001		< 0.001		< 0.001	
Per SD increase	1.25 (1.21–1.29)	< 0.001	1.18 (1.14–1.23)	< 0.001	1.13 (1.09–1.18)	< 0.001
TyG-WC						
Q1	Reference		Reference		Reference	
Q2	2.06 (1.79–2.38)	< 0.001	1.19 (1.03–1.38)	0.02	1.13 (0.98–1.31)	0.1
Q3	3.19 (2.79–3.65)	< 0.001	1.38 (1.2–1.59)	< 0.001	1.25 (1.09–1.45)	0.002
Q4	4.86 (4.27–5.53)	< 0.001	1.86 (1.62–2.14)	< 0.001	1.58 (1.37–1.82)	< 0.001
*P* for trend	< 0.001		< 0.001		< 0.001	
Per SD increase	1.61 (1.56–1.66)	< 0.001	1.28 (1.23–1.33)	< 0.001	1.21 (1.16–1.26)	< 0.001
TyG-WHtR						
Q1	Reference		Reference		Reference	
Q2	1.8 (1.58–2.05)	< 0.001	1.09 (0.95–1.24)	0.021	1.04 (0.91–1.19)	0.57
Q3	2.59 (2.29–2.93)	< 0.001	1.28 (1.13–1.45)	< 0.001	1.15 (1.02–1.31)	0.027
Q4	3.42 (3.03–3.85)	< 0.001	1.64 (1.45–1.85)	< 0.001	1.38 (1.22–1.56)	< 0.001
*P* for trend	< 0.001		< 0.001		< 0.001	
Per SD increase	1.44 (1.39–1.49)	< 0.001	1.22 (1.18–1.27)	< 0.001	1.15 (1.1–1.19)	< 0.001

AAD, aortic aneurysm and dissection; Model 1 has no variables adjusted; Model 2 adjusted age, sex, and race; Model 3 further adjusted with physical activity, smoking and drinking status, diet score, TDI, fasting time, family history of CVD, usage of antihypertensive drugs, lipid-lowering drugs, and insulin, cancer, hypertension, and diabetes.

**Figure 3:**
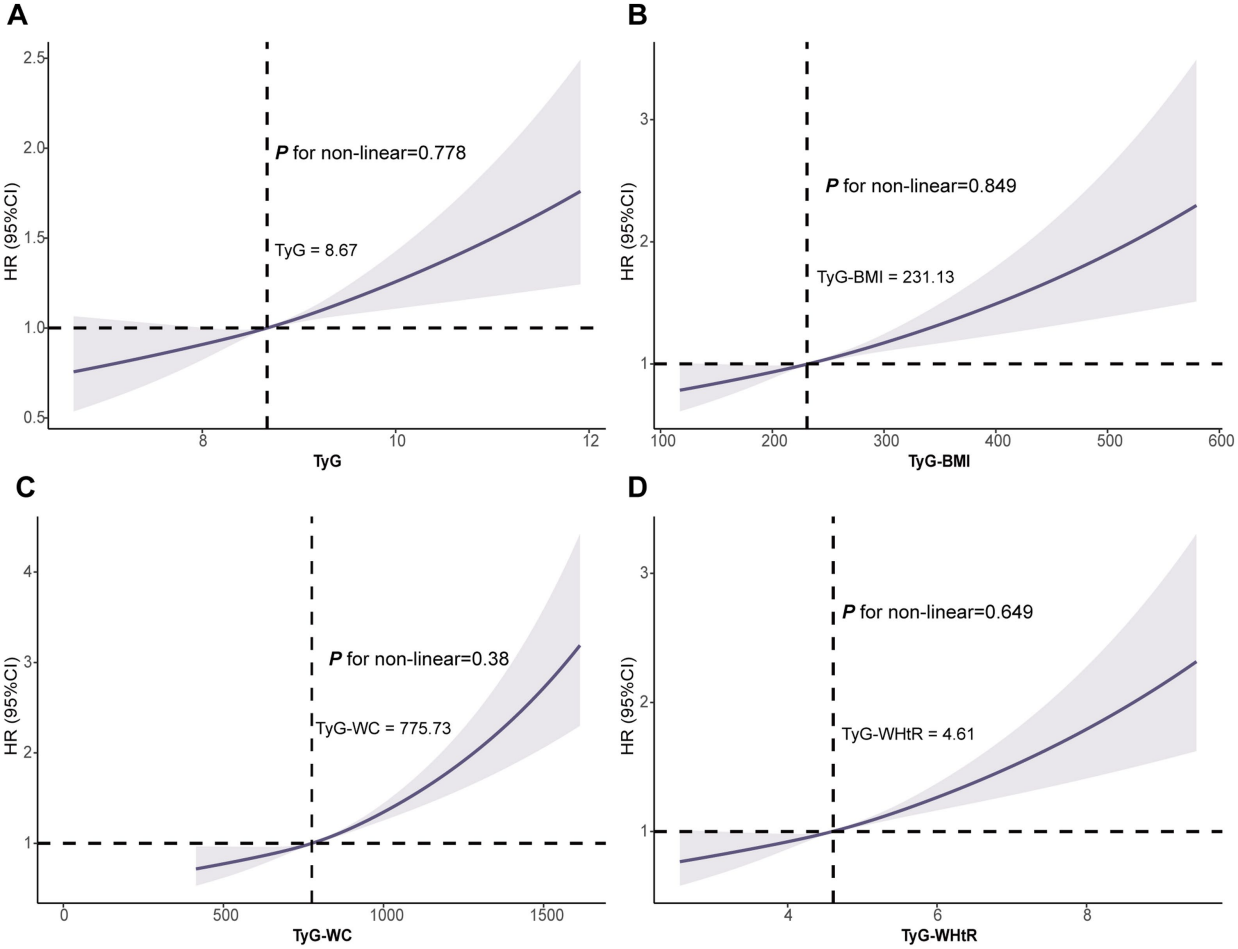
Association of the TyG **(A)**, TyG-BMI **(B)**, TyG-WC **(C)** and TyG-WHtR **(D)** with AAD using RCS. Models were fully adjusted with the maximum covariates in Model2. AAD, aortic aneurysm and dissection; RCS, Restricted cubic splines.

Subsequent RCS exhibited a linear dose-dependent relationship between all four indicators and AAD risk (all P for nonlinear >0.05; Figure 3).

### Impact of TyG, TyG-BMI, TyG-WC and TyG-WHtR on time to AAD onset

**Figure 4:**
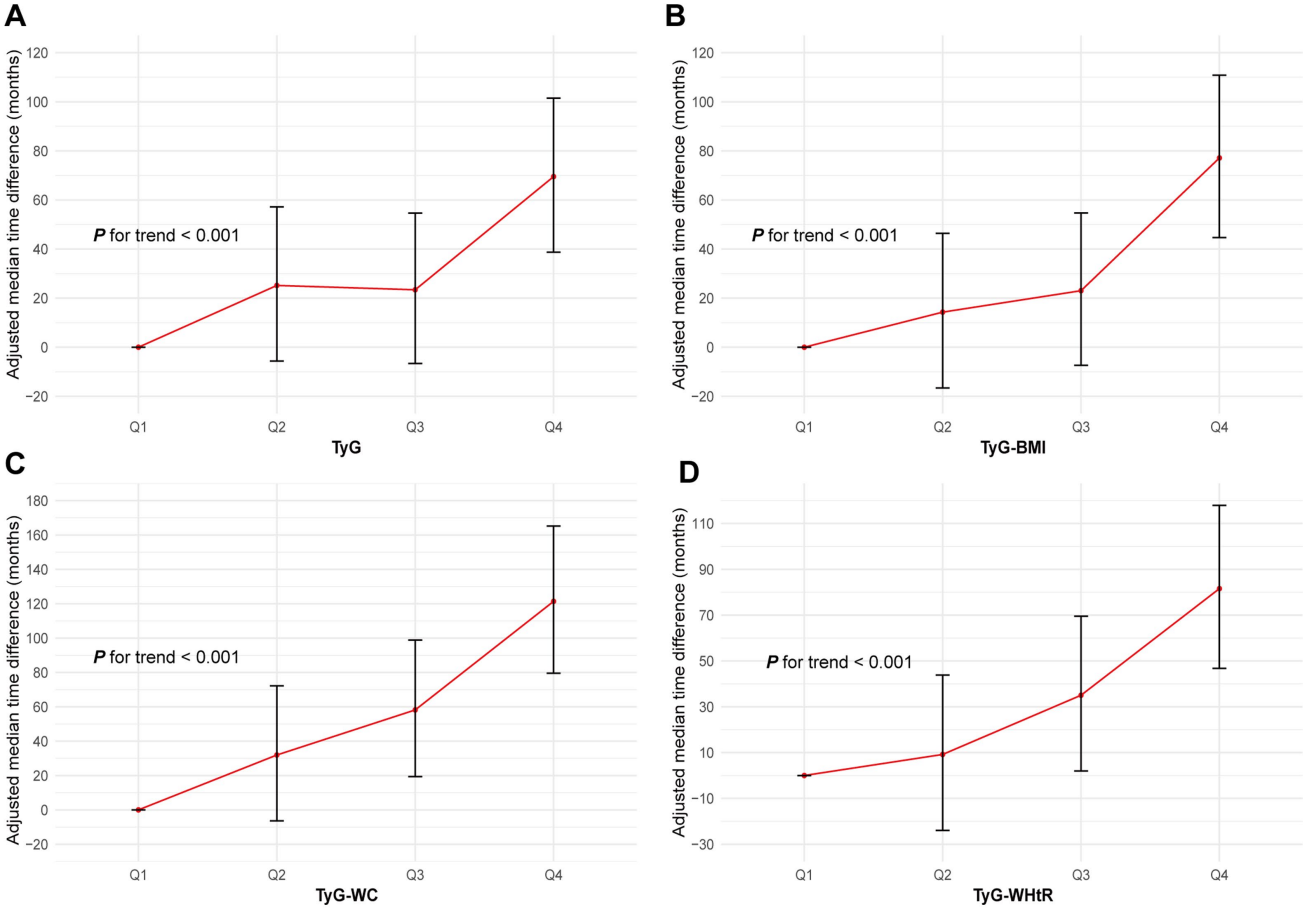
Association of the TyG **(A)**, TyG-BMI **(B)**, TyG-WC **(C)** and TyG-WHtR **(D)** with AAD using AFT. Models were fully adjusted with the maximum covariates in Model2. AAD, aortic aneurysm and dissection; TyG index, triglyceride glucose index; TyG-BMI, triglyceride glucose index–body mass index; TyG-WC, triglyceride glucose index-waist circumference; TyG-WHtR, triglyceride glucose index-waist height ratio; AFT, accelerated failure time.

AFT analysis revealed a decreasing time to AAD onset with increasing quartiles of TyG, TyG-BMI, TyG-WC and TyG-WHtR (all P for trend <0.05; Figure 4). Specifically, compared to the Q1 group, the median time to AAD onset in the Q2 to Q4 groups of the TyG index was advanced by 25.2 months, 54.6 months and 69.5 months, respectively. Similar trends were observed for TyG-BMI, TyG-WC and TyG-WHtR, particularly for TyG-WC, with the median time to AAD onset in the Q2 to Q4 groups advanced by 31.9 months, 58.2 months and 121.4 months, respectively (all P for trend <0.05; Figure 4; Supplementary Table S2).

Additionally, the ROC curve highlighted TyG-WC as the strongest predictor of AAD risk, with the highest AUC (AUC = 0.65, 95%CI: 0.64–0.66), followed by TyG-WHtR (AUC = 0.62, 95%CI: 0.61–0.63), TyG index (AUC = 0.59, 95%CI: 0.58–0.60) and TyG-BMI (AUC = 0.58, 95%CI: 0.57–0.59; Supplementary Figure S2). Notably, these results remained consistent across both the training and testing sets.

### Subgroup analyses

**Figure 5:**
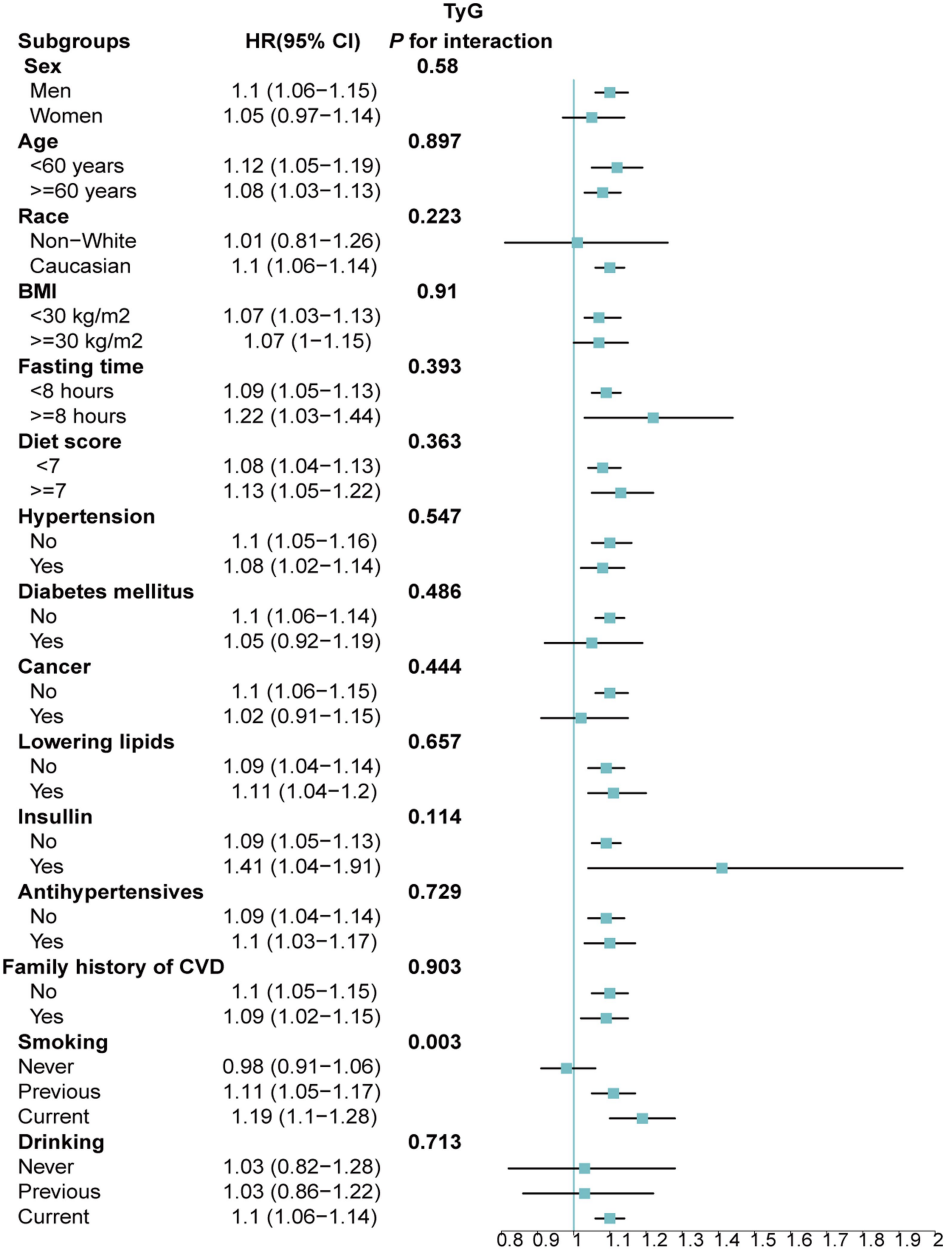
Association between each standard deviation increase in TyG and the risk of AAD stratified by different clinical characteristics. AAD, aortic aneurysm and dissection; TyG index, triglyceride glucose index.

**Figure 6:**
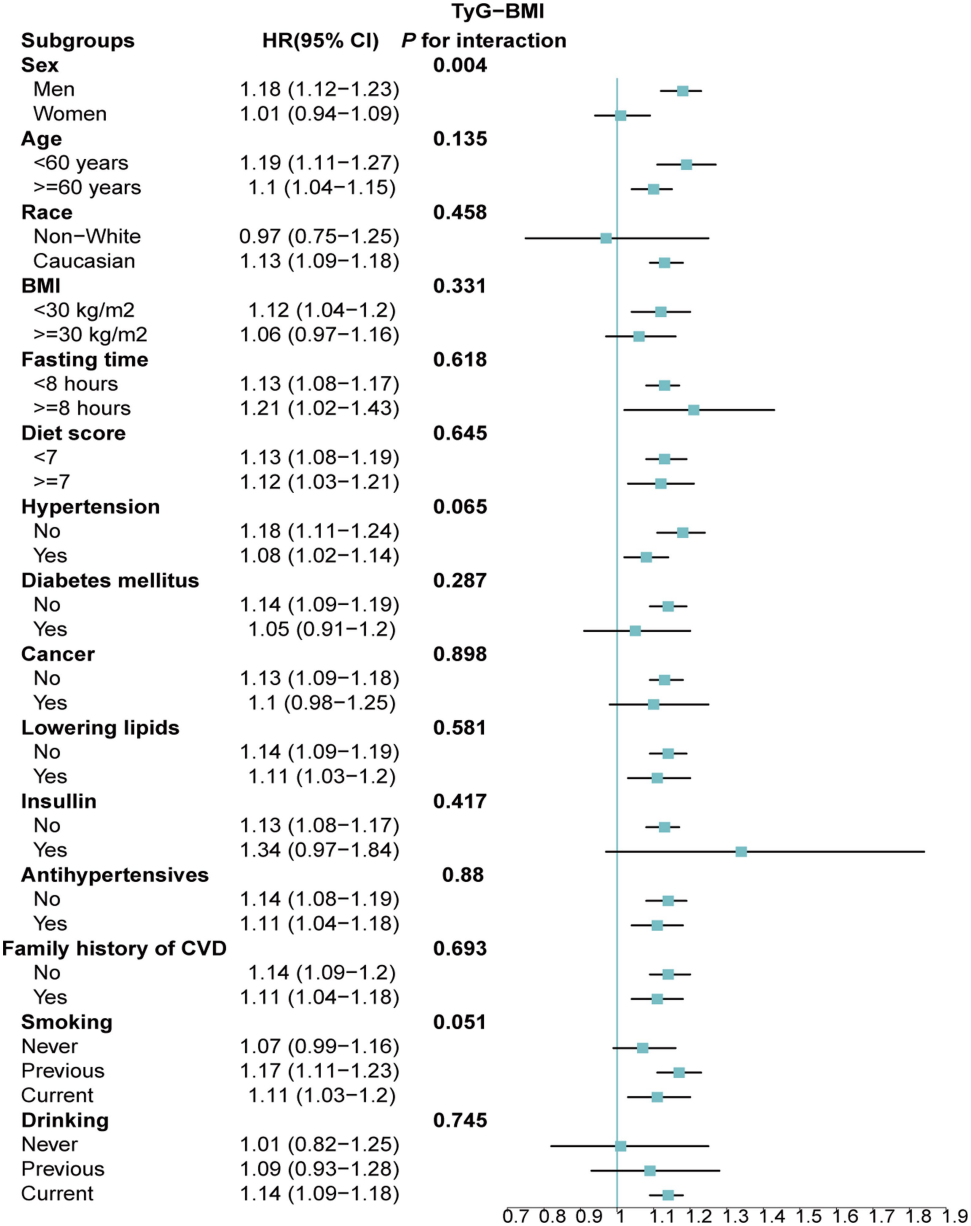
Association between each standard deviation increase in TyG-BMI and the risk of AAD stratified by different clinical characteristics. AAD, aortic aneurysm and dissection; TyG-BMI, triglyceride glucose index–body mass index.

**Figure 7:**
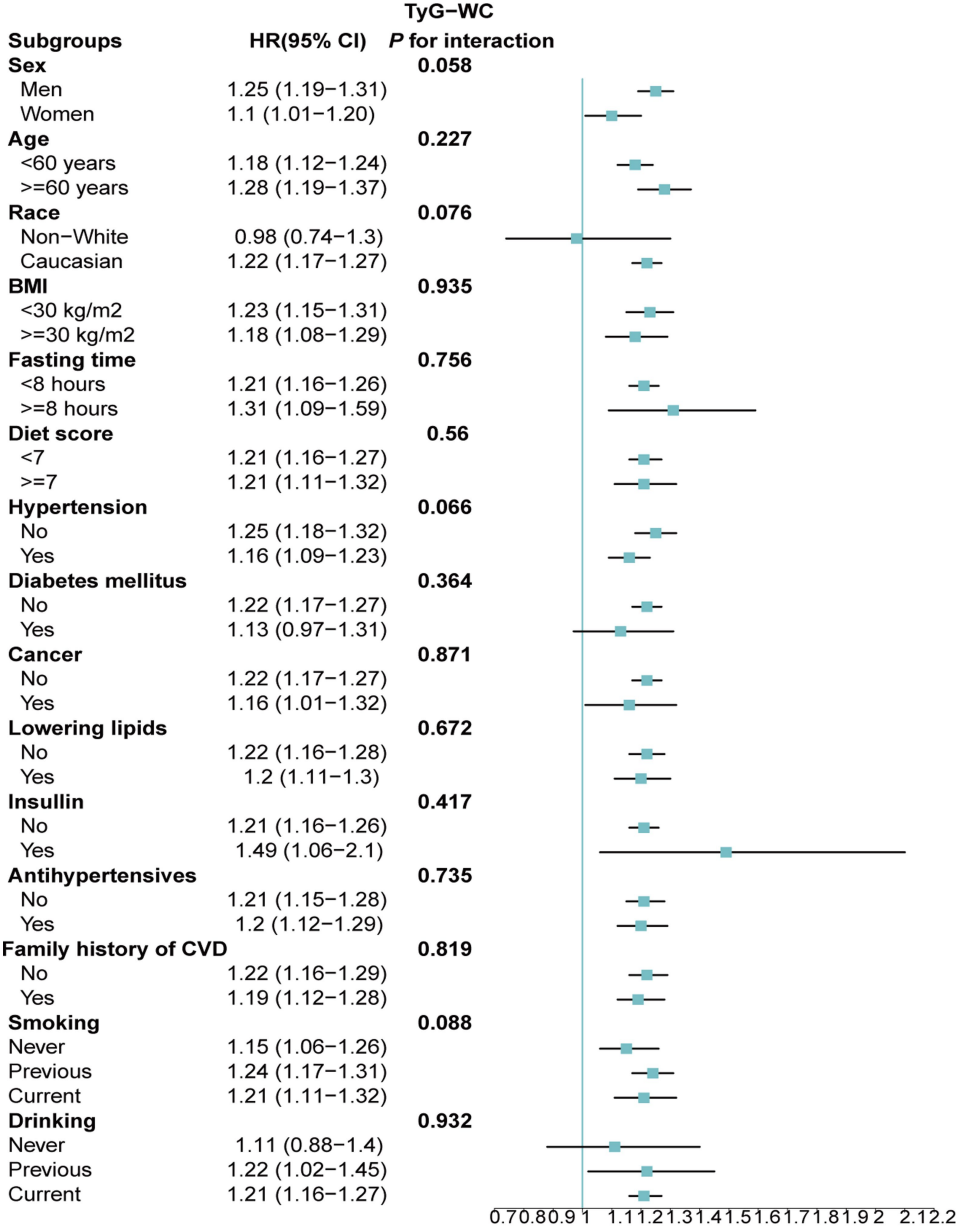
Association between each standard deviation increase in TyG-WC and the risk of AAD stratified by different clinical characteristics. AAD, aortic aneurysm and dissection; TyG-WC, triglyceride glucose index-waist circumference.

**Figure 8:**
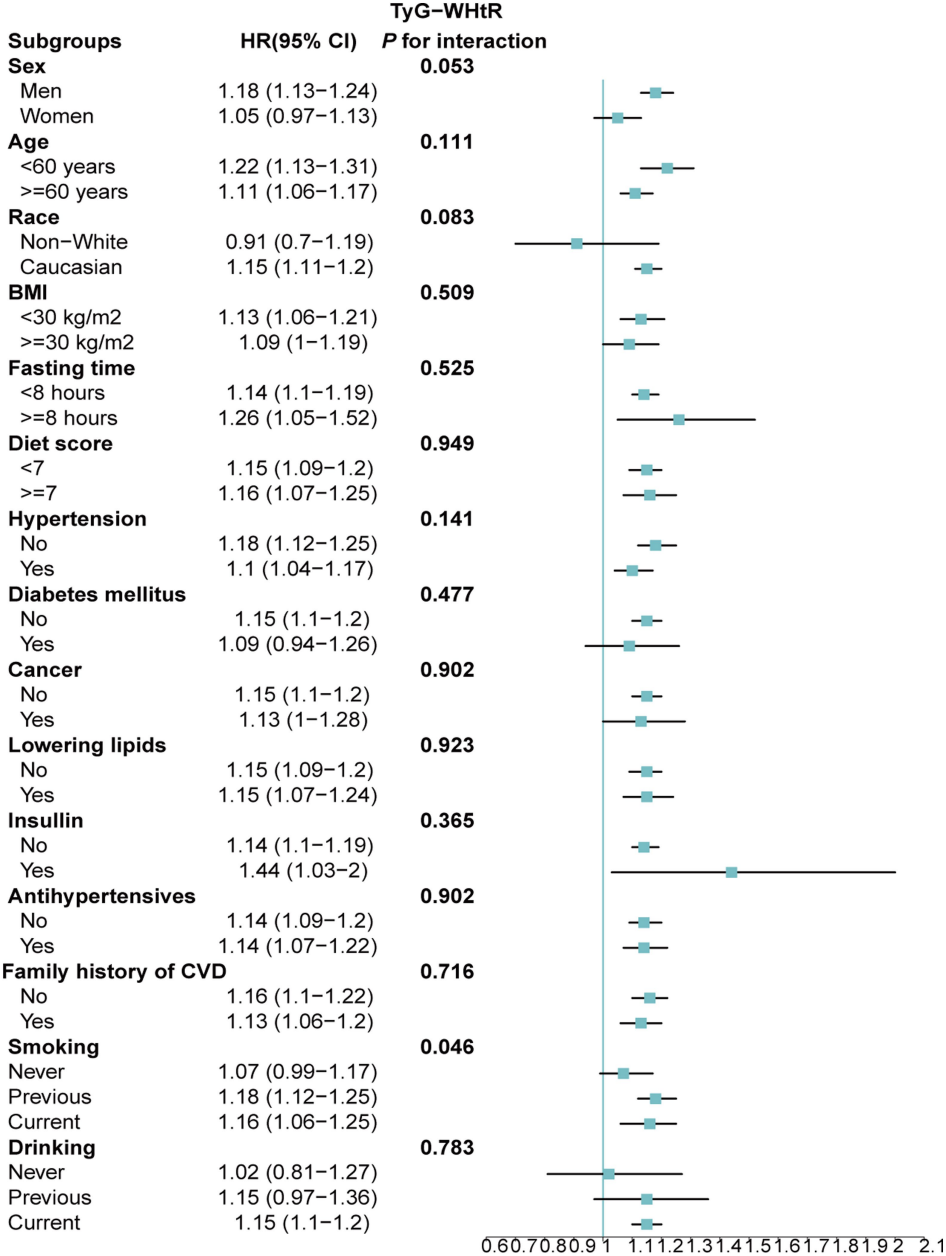
Association between each standard deviation increase in TyG-WHtR and the risk of AAD stratified by different clinical characteristics. AAD, aortic aneurysm and dissection; TyG-WHtR, triglyceride glucose index-waist height ratio.

Stratified analyses upheld the positive correlation between TyG, TyG-BMI, TyG-WC, TyG-WHtR and AAD risk across various subgroups, including age, medication use, diet score, family history of CVD and hypertension (Figures 5–8). Additionally, the association was more pronounced in male participants, individuals with BMI < 30 kg/m^2^, Caucasians, current smokers, alcohol consumers, and those without a history of cancer. Furthermore, significant interactions were noted between TyG indices and AAD risk, particularly in relation to smoking status for TyG (P for interaction = 0.003; Figure 5) and gender for TyG-BMI (P for interaction = 0.004; Figure 6).

### Sensitivity analyses

In sensitivity analyses, the exclusion of participants within 2 years of follow-up and those with missing baseline covariates yielded consistent results with the main findings (Supplementary Tables S3, S4). Additionally, PSM analysis effectively balanced baseline characteristics between AAD and non-AAD groups (Supplementary Table S5). Subsequent Cox proportional hazards models post-PSM adjustment demonstrated consistent results (Supplementary Table S6).

## Discussion

This large-scale prospective cohort study, to the best of our knowledge, represents the first investigation into the interplay between the TyG index, obesity indices and AAD risk. Our findings underscore a significant positive association between TyG, TyG-BMI, TyG-WC, TyG-WHtR and AAD risk, with a linear relationship observed. Furthermore, elevations in these indices significantly accelerated AAD occurrence. Among these indicators, TyG-WC exhibited the strongest association with AAD risk, as indicated by a larger AUC. Furthermore, the associations of the four indicators with AAD were particularly prominent among individuals with BMI < 30 kg/m^2^, Caucasians, current smokers, alcohol consumers and those without a history of cancer.

The TyG index, serving as a surrogate marker for IR, has garnered attention owing to its convenience and high sensitivity and specificity (37). In a retrospective cohort study of individuals over 40 years old, Hong S et al. reported a 26% increased risk of stroke (HR: 1.26, 95% CI: 1.23–1.29) and a 31% increased risk of myocardial infarction (HR: 1.31, 95% CI: 1.28–1.35) among participants in the TyG Q4 group compared to the Q1 group (37, 38). Similarly, Wan Y et al. demonstrated a linear association between each unit increase in TyG and a 16% increase in CVD risk, consistent with our findings (39). Conversely, Che B et al. identified a nonlinear relationship between TyG and CVD risk (40).

TyG-BMI, TyG-WC and TyG-WHtR represent combinations of TyG with obesity metrics. A prospective cohort study revealed that each SD increase in TyG-BMI correlated with a 17% increase in CVD risk (HR: 1.17, 95% CI: 1.04–1.31). The linear relationship between TyG-BMI and CVD risk observed in this study aligns with our findings (41). Another subgroup cohort study investigating the association between TyG-BMI and prehypertension (pre-HTN) or hypertension (HTN) identified TyG-BMI as an independent risk factor for the development of pre-HTN and HTN, with a linear correlation between TyG-BMI and pre-HTN/HTN risk, particularly showing significant sex interaction (42). Consistent with our study, we also observed a significant sex interaction in the relationship between TyG-BMI and AAD risk.

TyG-WC and TyG-WHtR are two additional indices utilized in CVD identification. Dang K et al. demonstrated that elevated levels of TyG-WC and TyG-WHtR significantly increase the risk of CVD (24, 37). Furthermore, another study indicated that TyG-WC and TyG-WHtR exhibit a linear relationship with developing CVD risk, with TyG-WC (AUC = 0.63) and TyG-WHtR (AUC = 0.65) outperforming TyG (AUC = 0.59) and TyG-BMI (AUC = 0.58) in predicting CVD risk (43). Similarly, Miao H et al. found that TyG-WC and TyG-WHtR surpass TyG and TyG-BMI in predicting CVD, with TyG-WC showing the strongest predictive capability (44). In our study, we similarly observed that TyG-WC and TyG-WHtR were closely associated with AAD risk, displaying a linear relationship. Notably, TyG-WC exhibited the highest predictive performance for AAD, followed by TyG-WHtR, TyG and TyG-BMI. Although the AUC of TyG-WC was 0.65, it should be emphasized that this is only the predictive ability of a single indicator. The clinical symptoms of AAD are highly variable and the etiology of the disease is complex, which poses a diagnostic challenge. The accuracy of any single biomarker in predicting AAD is limited. In the future, the combination of TyG-WC with other markers should be considered to improve the predictive ability of AAD.

AAD represents a challenging medical event to predict in advance (45). Although factors such as male gender, older age, hypertension and a family history of aneurysms are associated with AAD risk, identifying high-risk populations remains difficult due to its low incidence rate (8). The occurrence of AAD may be linked to various cardiovascular-related conditions (46). In our study, to eliminate the potential confounding effects of heart disease on the study results, participants with a history of heart disease at baseline were excluded. Subgroup analysis revealed that TyG, TyG-BMI, TyG-WC and TyG-WHtR demonstrated a stronger impact on AAD occurrence in men, Caucasians, individuals with BMI < 30 kg/m^2^, those without hypertension, diabetes, a history of cancer, and without a family history of cardiovascular disease.

The mechanisms underlying AAD development in relation to the TyG index and its derivatives, TyG-BMI, TyG-WC and TyG-WHtR, remain incompletely understood but likely involve several aspects. Firstly, the TyG index comprises lipid and glucose components. The lipid portion inhibits insulin secretion, leading to ectopic fat deposition in muscle cells and subsequent IR (47). The glucose component may elevate reactive oxygen species (ROS) levels, exerting toxic effects on pancreatic *β*-cells and impairing their function, thereby contributing to IR (48). TyG-BMI, TyG-WC and TyG-WHtR combined with obesity indicators, reflect the accumulation of visceral fat, further exacerbating IR and metabolic disturbances, which in turn mediate systemic inflammation, endothelial dysfunction and vascular remodeling, thereby promoting atherosclerosis (49). Previous studies have highlighted a strong correlation between the TyG index and atherosclerosis (50, 51). Atherosclerosis weakens the arterial wall, rendering it more susceptible to AAD under fluctuations in blood pressure or mechanical stress (52). Endothelial injury and inflammatory reactions further weaken the arterial wall, facilitating blood infiltration into the medial layer, ultimately resulting in AAD occurrence (52). Moreover, vascular endothelial secretion of inflammatory factors such as vascular cell adhesion molecule-1 (VCAM-1) and intercellular adhesion molecule-1 (ICAM-1) induces platelet adhesion and aggregation, promoting thrombus formation and vascular endothelial damage and increases the risk of dissection formation and extension (53–55).

Our study boasts certain strengths. First, it is the first to explore the relationship between TyG and obesity-related indicators and the AAD risk using a prospective approach and comprehensive long-term follow-up data. Additionally, subgroup analysis identified high-risk populations for AAD, and sensitivity analysis enhanced the robustness of the results. However, several limitations should also be acknowledged. Firstly, data on TyG, TyG-BMI, TyG-WC and TyG-WHtR were collected only at baseline, preventing observation of dynamic changes during follow-up and their impact on AAD. Secondly, despite adjusting for known confounding factors, unmeasured variables may still influence outcomes due to the observational study design, precluding the establishment of causality. Thirdly, our study’s predominantly middle-aged and older adults, along with a predominantly White population, may limit generalizability to other demographics. Lastly, the likelihood of healthy individuals participating during UKB recruitment may underestimate AAD incidence.

## Conclusion

The present study, based on a large prospective cohort design, showed that higher TyG index and its combination with obesity indices were significantly associated with the risk of AAD. Moreover, AFT models further showed that elevation of these indicators significantly advanced the onset of AAD. In addition, RCS analyses demonstrated a linear association between these indicators and the risk of AAD, and the TyG-WC showed higher predictive ability for AAD. These findings emphasize the potential application of the TyG index and its combination with obesity indicators in the early identification of AAD.

## Data Availability

The datasets presented in this study can be found in online repositories. The names of the repository/repositories and accession number(s) can be found in the article/Supplementary material.

## Footnotes

^1^ http://www.dagitty.net
