# Supplementary material for: Association of triglyceride-glucose index and its combination with obesity indicators in predicting the risk of aortic aneurysm and dissection
Source: Front Nutr. 2024 Oct 23;11:1454880. doi: 10.3389/fnut.2024.1454880 (PMC11537997; doi:10.3389/fnut.2024.1454880)
Supplement: Supplementary file 1 [file Data_Sheet_1.PDF]

Association of Triglyceride-Glucose Index and Its Combination with Obesity Indicators in Predicting the Risk of Aortic Aneurysm and Dissection

Table S1. Diagnostic codes for diseases 2

Table S2. Adjusted median time difference for AAD occurrence in quintile groups 3

Table S3. The association between TyG, TyG-BMI, TyG-WC, TyG-WHtR and the risk of AAD after excluding participants within two years of follow-up 4

Table S4. The association between TyG, TyG-BMI, TyG-WC, TyG-WHtR and the risk of AAD after excluding any missing value at baseline 5

Table S5. Baseline demographic and clinical characteristics after PSM 6

Table S6. The association between TyG, TyG-BMI, TyG-WC, TyG-WHtR and the risk of AAD after PSM 7

Figure S1. Directed acyclic graph of the link between TyG ,TyG-BMI, TyG-WC and TyG-WHtR and the onset of AAD. 8

Figure S2. Receiver operating characteristic (ROC) curves of TyG, TyG-BMI, TyG-WC and TyG-WHtR in relation to AAD risk. 9

Table S1. Diagnostic codes for diseases

Diseases	ICD-9	ICD-10	Self-report
Coronary artery disease	410, 4109, 411, 4119, 412, 4129, 413, 4139, 4140, 4148, 4149	I20-I25	20002
Heart failure	428, 4280, 4281, 4289	I50	20002
Arrhythmias	426, 4260, 4261, 4263, 4264, 4265, 4266, 4267, 4269, 427, 4270, 4271, 4273, 4274, 4276, 4278, 4279	I44, I45, I47-I49	20002
Cardiomyopathy	422, 4229, 425, 4251, 4254	I40-I43	20002
Heart valve disease	394, 3940, 3942, 3949, 395, 3951, 3959, 396, 3969, 424, 4240, 4241, 4243, 4249	I05-I09, I34-I38	20002

ICD: International Classification of Disease.

Table S2. Adjusted median time difference for AAD occurrence in quintile groups

Categories	Median difference (months)	95% Lower CI	95% Upper CI
	TyG		
Q1	Ref		
Q2	25.2	57.2	-5.6
Q3	23.4	-6.6	69.52
Q4	69.5	101.5	38.7
	TyG-BMI		
Q1	Ref		
Q2	14.3	46.4	-16.6
Q3	23	54.7	-7.4
Q4	77.1	110.8	44.7
	TyG-WC		
Q1	Ref		
Q2	31.9	72.2	-6.4
Q3	58.2	98.8	19.4
Q4	121.4	165.2	79.6
	TyG-WHtR		
Q1	Ref		
Q2	9.2	43.9	-24
Q3	35	69.5	2
Q4	81.6	117.9	46.8

Models were fully adjusted with the maximum covariates in Model 3. AAD: aortic aneurysm and dissection**;** TyG index: triglyceride glucose index; TyG-BMI: triglyceride glucose index–body mass index; TyG-WC: triglyceride glucose index-waist circumference; TyG-WHtR: triglyceride glucose index-waist height ratio;

Table S3. The association between TyG, TyG-BMI, TyG-WC, TyG-WHtR and the risk of AAD after excluding participants within two years of follow-up

Type	HR (95%CI)	P
**TyG**		
Q1	Reference	
Q2	1.13 (1-1.27)	0.055
Q3	1.1 (0.98-1.24)	0.106
Q4	1.32 (1.17-1.48)	<0.001
P for trend	< 0.001	
Per SD increase	1.1 (1.06-1.14)	< 0.001
**TyG-BMI**		
Q1	Reference	
Q2	1.08 (0.95-1.22)	0.247
Q3	1.14 (1.01-1.28)	0.034
Q4	1.4 (1.24-1.57)	<0.001
P for trend	< 0.001	
Per SD increase	1.14 (1.09-1.19)	< 0.001
**TyG-WC**		
Q1	Reference	
Q2	1.12 (0.96-1.3)	0.146
Q3	1.27 (1.09-1.47)	0.002
Q4	1.59 (1.37-1.84)	<0.001
P for trend	< 0.001	
Per SD increase	1.22 (1.17-1.27)	< 0.001
**TyG-WHtR**		
Q1	Reference	
Q2	1.04 (0.91-1.2)	0.529
Q3	1.18 (1.04-1.35)	0.011
Q4	1.41 (1.24-1.6)	<0.001
P for trend	< 0.001	
Per SD increase	1.15 (1.11-1.2)	< 0.001

Models were fully adjusted with the maximum covariates in Model 3. AAD: aortic aneurysm and dissection; TyG index: triglyceride glucose index; TyG-BMI: triglyceride glucose index–body mass index; TyG-WC: triglyceride glucose index-waist circumference; TyG-WHtR: triglyceride glucose index-waist height ratio;

Table S4. The association between TyG, TyG-BMI, TyG-WC, TyG-WHtR and the risk of AAD after excluding any missing value at baseline

**Type**	HR (95%CI)	P
**TyG**		
Q1	Reference	
Q2	1.08 (0.94-1.24)	0.268
Q3	1.04 (0.91-1.19)	0.548
Q4	1.24 (1.09-1.41)	<0.001
P for trend	< 0.001	
Per SD increase	1.09 (1.04-1.13)	< 0.001
**TyG-BMI**		
Q1	Reference	
Q2	1.03 (0.9-1.19)	0.642
Q3	1.08 (0.95-1.24)	0.252
Q4	1.37 (1.2-1.57)	<0.001
P for trend	< 0.001	
Per SD increase	1.14 (1.09-1.19)	< 0.001
**TyG-WC**		
Q1	Reference	
Q2	1.17 (0.98-1.39)	0.075
Q3	1.23 (1.04-1.46)	0.017
Q4	1.59 (1.35-1.89)	<0.001
P for trend	< 0.001	
Per SD increase	1.22 (1.16-1.28)	< 0.001
**TyG-WHtR**		
Q1	Reference	
Q2	1 (0.86-1.16)	0.996
Q3	1.06 (0.91-1.23)	0.452
Q4	1.36 (1.18-1.57)	<0.001
P for trend	< 0.001	
Per SD increase	1.15 (1.1-1.21)	< 0.001

Models were fully adjusted with the maximum covariates in Model 3. AAD: aortic aneurysm and dissection**;** TyG index: triglyceride glucose index; TyG-BMI: triglyceride glucose index–body mass index; TyG-WC: triglyceride glucose index-waist circumference; TyG-WHtR: triglyceride glucose index-waist height ratio;

Table S5. Baseline demographic and clinical characteristics after PSM

Characteristic	Total (n=5988)	Non-AAD (n=2994)	AAD (n=2994)	*P*-value
Age, years	63.0 (58.0-66.0)	63.0 (59.0-66.0)	63.0 (58.0-66.0)	0.489
Male	4561 (76.2%)	2280 (76.2%)	2281 (76.2%)	0.976
White	5831 (97.4%)	2911 (97.2%)	2920 (97.5%)	0.467
MET	1850.5 (810.0-3799.5)	1862.0 (850.0-3816.0)	1828.0 (744.6-3786.0)	0.109
TDI	-2.2 (-3.7-0.5)	-2.2 (-3.7-0.6)	-2.2 (-3.6-0.5)	0.676
Fasting time	3.0 (3.0-5.0)	3.0 (3.0-5.0)	3.0 (3.0-4.0)	0.557
Diet score	5.0 (4.0-6.0)	5.0 (4.0-6.0)	5.0 (4.0-6.0)	0.802
DM	331 (5.5%)	161 (5.4%)	170 (5.7%)	0.611
Hypertension	2602 (43.5%)	1310 (43.8%)	1292 (43.2%)	0.639
Cancer	618 (10.3%)	316 (10.6%)	302 (10.1%)	0.552
History family of CVD	2316 (38.7%)	1150 (38.4%)	1166 (38.9%)	0.671
Lipid-lowering drugs	1555 (26.0%)	766 (25.6%)	789 (26.4%)	0.498
Antihypertensives	2019 (33.7%)	1011 (33.8%)	1008 (33.7%)	0.935
Insulin	34 (0.6%)	13 (0.4%)	21 (0.7%)	0.169
Drinking status				0.921
Never	163 (2.7%)	84 (2.8%)	79 (2.6%)	
Previous	263 (4.4%)	132 (4.4%)	131 (4.4%)	
Current	5562 (92.9%)	2778 (92.8%)	2784 (93.0%)	
Smoking status				0.447
Never	1504 (25.1%)	770 (25.7%)	734 (24.5%)	
Previous	3150 (52.6%)	1552 (51.8%)	1598 (53.4%)	
Current	1334 (22.3%)	672 (22.4%)	662 (22.1%)	
TyG	8.8 (8.5-9.2)	8.8 (8.4-9.2)	8.8 (8.5-9.2)	<0.001
TyG-BMI	242.5 (215.1-275.1)	239.6 (213.5-271.7)	245.5 (217.2-277.9)	<0.001
TyG-WC	844.2 (754.0-935.8)	833.5 (746.1-920.5)	854.9 (761.8-948.7)	<0.001
TyG-WHtR	4.9 (4.4-5.4)	4.8 (4.4-5.4)	4.9 (4.4-5.5)	<0.001

BMI: body mass index; WC: waist circumference; MET: metabolic equivalent task; TDI: Townsend deprivation index; DM: diabetes mellitus.

Table S6. The association between TyG, TyG-BMI, TyG-WC, TyG-WHtR and the risk of AAD after PSM

**Type**	HR (95%CI)	P
**TyG**		
Q1	Reference	
Q2	1.03 (0.93-1.15)	0.537
Q3	1.08 (0.97-1.2)	0.158
Q4	1.19 (1.08-1.32)	<0.001
P for trend	< 0.001	
Per SD increase	1.06 (1.02-1.1)	0.002
**TyG-BMI**		
Q1	Reference	
Q2	1 (0.9-1.12)	0.943
Q3	1.14 (1.03-1.27)	0.013
Q4	1.21 (1.09-1.35)	<0.001
P for trend	< 0.001	
Per SD increase	1.08 (1.04-1.12)	< 0.001
**TyG-WC**		
Q1	Reference	
Q2	1.05 (0.94-1.17)	0.412
Q3	1.2 (1.08-1.34)	0.001
Q4	1.36 (1.22-1.53)	<0.001
P for trend	< 0.001	
Per SD increase	1.15 (1.1-1.19)	< 0.001
**TyG-WHtR**		
Q1	Reference	
Q2	1 (0.9-1.11)	0.991
Q3	1.14 (1.03-1.27)	0.015
Q4	1.21 (1.08-1.34)	<0.001
P for trend	< 0.001	
Per SD increase	1.09 (1.05-1.14)	< 0.001

Models were fully adjusted with the maximum covariates in Model 3. AAD: aortic aneurysm and dissection**;** TyG index: triglyceride glucose index; TyG-BMI: triglyceride glucose index–body mass index; TyG-WC: triglyceride glucose index-waist circumference; TyG-WHtR: triglyceride glucose index-waist height ratio;

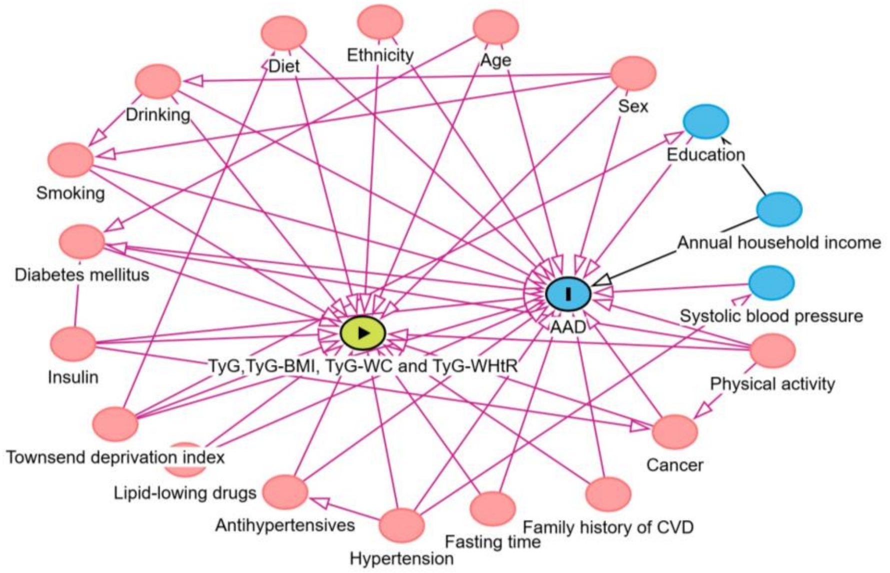

Figure S1. Directed acyclic graph of the link between TyG ,TyG-BMI, TyG-WC and TyG-WHtR and the onset of AAD.

Diagram created with the help of DAGitty.net (www.dagitty.net). Minimal adjustment set: sex, age, race, TDI, fasting time, physical activity, diet score, diabetes, lowering lipids, antihypertensives, insulin, smoking and drinking status, hypertension, cancer and DM.

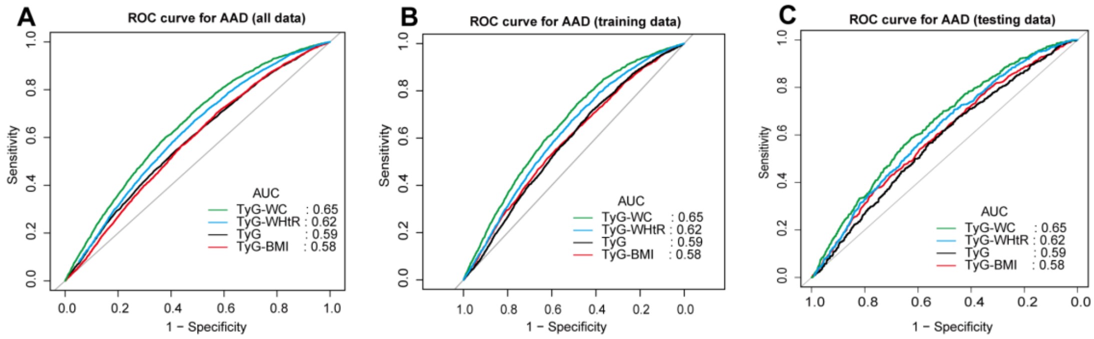

Figure S2. Receiver operating characteristic (ROC) curves of TyG, TyG-BMI, TyG-WC and TyG-WHtR in relation to AAD risk. AAD: aortic aneurysm and dissection; TyG index: triglyceride glucose index; TyG-BMI: triglyceride glucose index–body mass index; TyG-WC: triglyceride glucose index-waist circumference; TyG-WHtR: triglyceride glucose index-waist height ratio.
